# Melanoma of the Scalp Harboring a Novel *AGFG1* :: *ERBB4* Fusion: A Case Report With Clinicopathologic and Genomic Characterization

**DOI:** 10.1002/gcc.70183

**Published:** 2026-10-28

**Authors:** Xu Cao, Hong Yu, Kenneth L. Muldrew, Rachael M. Johnson, Li Liang, Ying Pei, Yve T. Huttenbach, C. Cameron Yin, Songlin Zhang

**Affiliations:** ^1^ Department of Pathology & Immunology Baylor College of Medicine Houston Texas USA; ^2^ Department of Pathology and Laboratory Medicine Ben Taub Hospital, Harris Health System Houston Texas USA; ^3^ School of Health Professions University of Texas MD Anderson Cancer Center Houston Texas USA; ^4^ Department of Pathology and Laboratory Medicine Michael E. DeBakey Veterans Affairs Medical Center Houston Texas USA; ^5^ Section of Infectious Diseases, Department of Medicine Baylor College of Medicine Houston Texas USA; ^6^ School of Medicine Baylor College of Medicine Houston Texas USA; ^7^ Department of Hematopathology The University of Texas M.D. Anderson Cancer Center Houston Texas USA

**Keywords:** *AGFG1::ERBB4*, *ERBB4* fusion, kinase rearrangement, melanoma

## Abstract

*ERBB4* (HER4) gain‐of‐function missense mutations have been implicated in cutaneous melanoma. In contrast, structural rearrangements involving *ERBB4* are exceedingly rare. We describe a 59‐year‐old man with a rapidly enlarging 4.2‐cm melanoma of the scalp with high‐risk pathologic features, including epithelioid to plasmacytoid cytomorphology, ulceration, brisk mitotic activity, and microscopic satellitosis. RNA sequencing identified a novel in‐frame *AGFG1*::*ERBB4* fusion joining *AGFG1* exon 2 to *ERBB4* exon 2. The *ERBB4* portion retains the downstream coding sequence, including the tyrosine kinase domain. Concurrent molecular alterations included a pathogenic *NF1* loss‐of‐function alteration, a *TERT* promoter mutation, *CDK4* and *STAT6* copy‐number gains, and *TP53* copy‐number loss in a *BRAF*/*NRAS*/*KIT*‐wild‐type background.

## 1 Introduction

Cutaneous melanoma is highly genomically heterogeneous and has one of the highest somatic mutational burdens among human cancers [1]. Current molecular classification of melanoma recognizes several categories defined by their distinct genomic landscapes: high‐CSD (chronically sun‐damaged) melanoma, low‐CSD melanoma, acral melanoma, mucosal melanoma, uveal melanoma, and melanoma arising in (or resembling) blue nevus—blue‐nevus‐like melanoma [2], together with Spitz melanoma and the newer category of melanoma arising from Wnt‐activated deep penetrating/plexiform melanocytoma or nevus [3]. Both lentigo maligna melanoma (LMM) and desmoplastic melanoma are categorized within the high‐CSD melanoma group.

Although targeted therapies have transformed outcomes for patients with *BRAF*‐mutant melanoma, effective molecularly guided treatment options for *BRAF*‐wild‐type disease remain comparatively limited [4]. Many *BRAF*‐wild‐type melanomas harbor activating *NRAS* mutations or loss‐of‐function *NF1* alterations that converge on RAS‐MAPK pathway activation; however, MEK inhibitor monotherapy and other strategies targeting downstream RAS‐MAPK signaling have shown limited clinical benefit in these settings [5]. These challenges highlight the need to identify additional actionable molecular alterations that may refine classification and therapeutic stratification of *BRAF*‐wild‐type melanoma. Gene fusions are rare in melanoma (approximately 2%) but may represent actionable therapeutic targets, supporting their evaluation in patients with advanced diseases who have progressed following immunotherapy [6].

ERBB4, also known as HER4, is a receptor tyrosine kinase within the ERBB/HER family, which also includes EGFR/ERBB1, HER2/ERBB2, and HER3/ERBB3 [7]. Activating *ERBB4* point mutations have been described in melanoma, are enriched in *BRAF*‐wild‐type tumors, and have been shown to increase kinase activity, promote transformation, and confer sensitivity to ERBB inhibition [8, 9, 10]. These alterations may promote tumorigenesis by activating downstream RAS‐MAPK and PI3K‐AKT signaling pathways. However, compared with point mutations, *ERBB4* structural rearrangements are rarely reported in melanoma, and their clinicopathologic significance remains poorly understood.

Here, we describe a novel *AGFG1::ERBB4* fusion identified in a scalp melanoma within a *BRAF/NRAS/KIT–wild‐type* and *NF1*‐mutant genomic background.

## 2 Case Presentation

A 59‐year‐old man presented with a rapidly enlarging, non‐tender lesion on the right parietal scalp. He initially noticed the lesion after shaving and attributed it to a small cut. The mass rapidly enlarged over the months prior to evaluation. Physical examination revealed an approximately 4.2 cm × 4.2 cm exophytic, thick, friable plaque with peripheral hemorrhagic crust and scant purulent drainage. No cervical, axillary, or inguinal lymphadenopathy was identified.

Computed tomography of the head demonstrated an exophytic soft‐tissue mass arising from the right parietal scalp without invasion into subcutaneous fat or calvarium and without intracranial abnormality. Punch biopsy revealed an ulcerated malignant neoplasm composed of epithelioid to plasmacytoid cells arranged in sheets and nests with high nuclear‐to‐cytoplasmic ratios, marked pleomorphism, intranuclear pseudoinclusions, prominent nucleoli, and brisk mitotic activity. Immunohistochemistry demonstrated diffuse positivity for S100, SOX10, HMB45, and MART‐1 with negativity for CAM5.2, AE1/AE3, and p40, confirming the diagnosis of melanoma (Figure 1).

**FIGURE 1:**
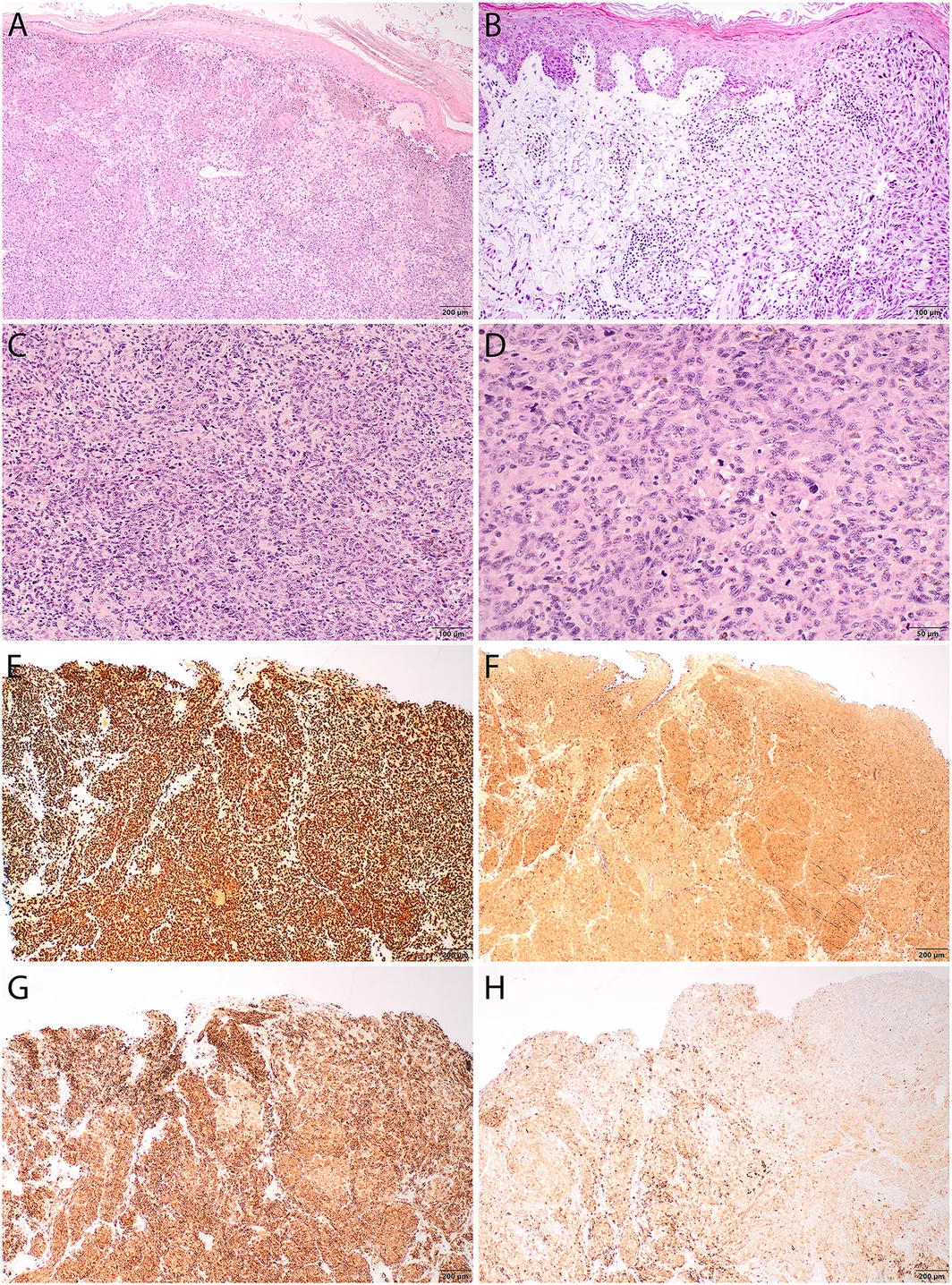
Histologic and immunohistochemical features of the scalp melanoma. (A–D) The tumor is composed of epithelioid to plasmacytoid cells exhibiting marked cytologic atypia, prominent nucleoli, and brisk mitotic activity. No peripheral extension of melanoma in situ is identified beyond the invasive component (B). (E–H) Immunohistochemical stains demonstrate diffuse positivity for SOX10 (E), S100 (F), MART‐1 (G), and HMB45 (H). (A, E–H, 40×; B and C, 100×; D, 200×).

The patient underwent wide local excision with sentinel lymph node excision and sampling of right external jugular lymph nodes. Pathologic examination of the excision specimen demonstrated invasive melanoma with a Breslow thickness of 15 mm and Clark Level V invasion into subcutis. Ulceration was present with an extent of 20 mm, and the mitotic rate was 13 mitoses/mm^2^. Lymphovascular and perineural invasion were not identified. Microscopic satellitosis was identified. Resection margins were negative, and 12 regional lymph nodes examined, including 10 sentinel nodes and 2 right external jugular lymph nodes, were negative for metastatic melanoma. The tumor was staged as pT4bN1c (Stage IIIC) according to the American Joint Committee on Cancer (AJCC) 8th edition melanoma staging criteria.

Given the aggressive clinical presentation and high‐risk pathologic features, comprehensive genomic profiling was performed using a clinically validated DNA‐ and RNA‐based next‐generation sequencing panel from Tempus (Chicago, IL, USA). RNA sequencing (xR) identified an in‐frame *AGFG1*::*ERBB4* fusion joining the end of *AGFG1* exon 2 to the start of *ERBB4* exon 2, retaining the ERBB4 tyrosine kinase domain (Figure 2). On the GRCh37/hg19 reference, the breakpoints were chr2:228356356 in *AGFG1* (NM**_**001135187.2, exon 2) and chr2:212989628 in *ERBB4* (NM**_**005235.3, exon 2). The fusion junction sequence is 5′‐[AGFG1] ATGGAAAATGAA | TGTGTGCAGGAA [ERBB4]‐3′, which translates to MENECVQE in the *AGFG1* reading frame, consistent with preservation of the open reading frame and retention of the downstream *ERBB4* coding sequence. The Tempus xR assay is a whole‐transcriptome, hybrid‐capture next‐generation sequencing (NGS) assay that detects chromosomal rearrangements and altered splicing events using custom‐designed IDT probes. The assay uses the IDT xGen Exome Research Panel v2 as its backbone, which comprises more than 415 000 individually synthesized probes targeting approximately 34 Mb across 19 433 genes. The post‐microdissection tumor content in this case was 70%. The coordinates chr2:228356356 (*AGFG1*) and chr2:212989628 (*ERBB4*) were derived from the RNA fusion pipeline as the inferred genomic positions of the transcript junction and were not independently observed as a DNA structural variant. Additionally, orthogonal validation was not performed, and the functional significance of this fusion remains to be established.

**FIGURE 2:**
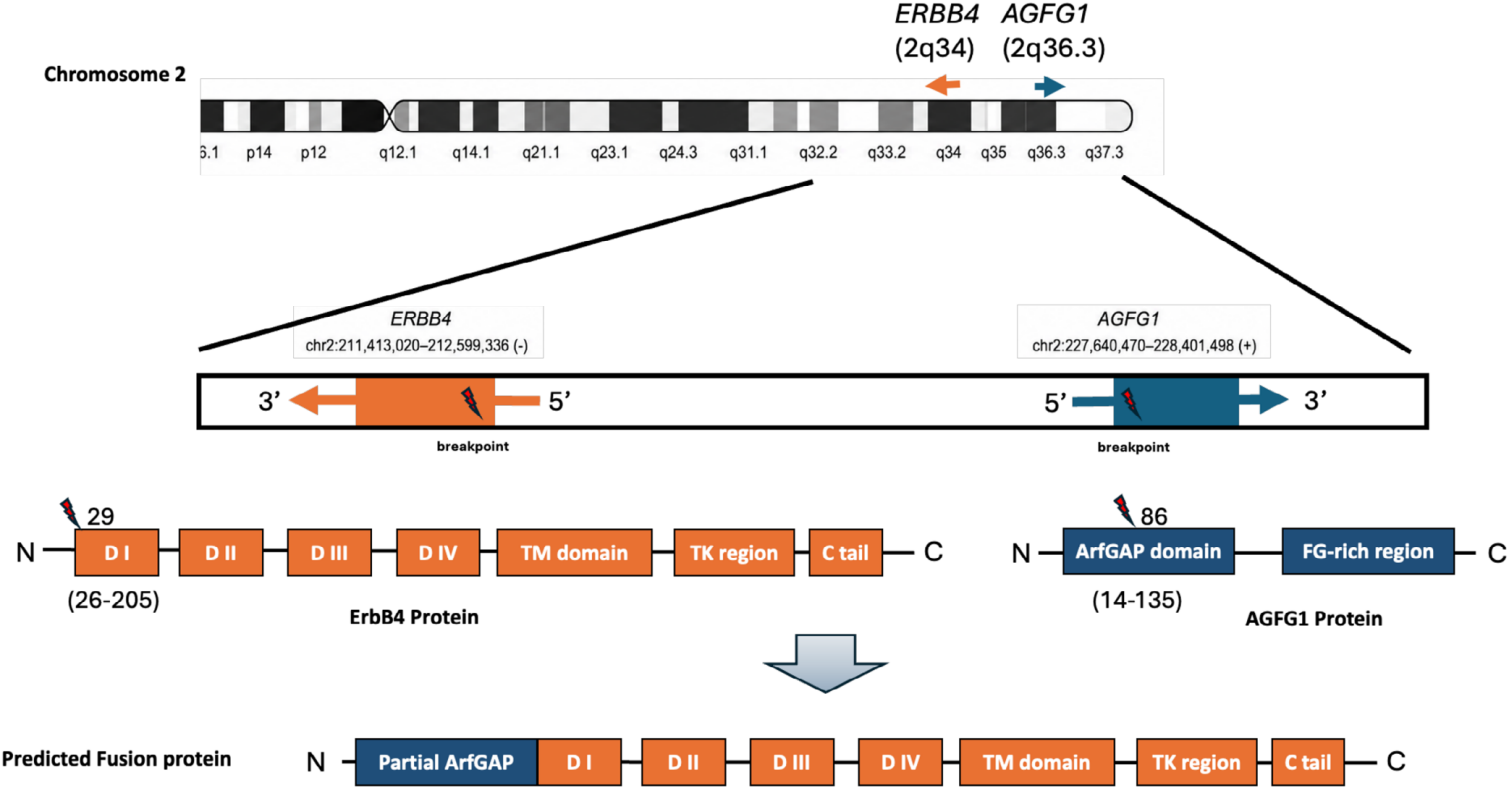
Schematic representation of the *AGFG1*::*ERBB4* fusion, showing the fusion of *AGFG1* exon 2 to *ERBB4* exon 2. The breakpoint lies within the N‐terminal ArfGAP domain of *AGFG1*; therefore, only a partial ArfGAP domain is predicted to be retained, while the remainder of the ArfGAP domain and the downstream FG‐rich region are not retained. The *ERBB4* contribution begins in exon 2, downstream of the native signal peptide, near the N‐terminal portion of extracellular domain I. The predicted fusion therefore retains near‐complete extracellular domain I, complete extracellular domains II–IV, the transmembrane region, the intracellular tyrosine kinase domain, and the C‐terminal signaling region.

DNA sequencing identified a tumor mutational burden of 15.3 mutations/Mb. Concurrent molecular alterations included an *NF1* splice‐site variant, c.1641 + 2T>G, predicted to result in loss of function; a *TERT* promoter mutation, c.‐146C>T; *CDK4* and *STAT6* copy‐number gains; and *TP53* copy‐number loss. The tumor was microsatellite stable and showed no pathogenic alterations in *BRAF*, *NRAS*, or *KIT*.

Although identification of the *ERBB4* fusion suggested a potential role for *ERBB*‐directed targeted therapy, tyrosine kinase inhibitor treatment was not pursued because this approach remains investigational in melanoma. The patient received standard adjuvant anti‐PD‐1 immunotherapy with pembrolizumab and had no evidence of recurrence at 15 months of follow‐up.

## 3 Discussion

The present case harbored an *NF1* loss‐of‐function alteration and a *TERT* promoter mutation, together with a high tumor mutational burden in a *BRAF*/*NRAS*/*KIT*–wild‐type background, which is consistent with a high‐CSD molecular profile [1]. This profile also corresponds to the *NF1*‐mutant molecular subtype in the TCGA four‐group framework, which denotes *BRAF*/*NRAS*–wild‐type tumors with high mutation burden arising in sun‐damaged head‐and‐neck skin of older patients [11].

Structural rearrangements involving *ERBB4* may activate receptor tyrosine kinase signaling across multiple tumor types, with the location of the fusion breakpoint determining the predicted structure of the chimeric protein and its mechanism of activation. One pattern comprises kinase‐only fusions, in which a 5′ partner is joined to the C‐terminal portion of ERBB4 containing the tyrosine kinase domain; examples include *EZR*::*ERBB4* in lung adenocarcinoma [12] and *PARD3B*::*ERBB4* reported in HPV‐associated adenosquamous carcinoma of the oropharynx [13]. A second pattern comprises full‐receptor (extracellular‐domain–retaining) fusions, in which breakpoints near the 5′ end of *ERBB4* preserve the extracellular, transmembrane, and intracellular tyrosine kinase domains; *IKZF2*::*ERBB4*, a recurrent event in peripheral T‐cell lymphoma, has been proposed as an example, although its precise breakpoint and domain architecture warrant confirmation [14].

The novel *AGFG1::ERBB4* fusion described here follows the receptor‐preserving pattern. This junction places the N‐terminal *AGFG1* segment upstream of a largely intact *ERBB4* receptor. *AGFG1* and *ERBB4* are both located on the long arm of chromosome 2 but in opposite transcriptional orientations, with *AGFG1* on the plus strand and *ERBB4* on the minus strand. Because the two genes are antiparallel, a simple interstitial deletion would not co‐orient the partners into a sense transcript; the observed in‐frame *AGFG1*::*ERBB4* fusion therefore most plausibly arises from an inversion or a more complex intrachromosomal rearrangement, and the exact genomic mechanism cannot be determined from RNA fusion data alone [15]. *AGFG1* encodes a phenylalanine‐glycine (FG) repeat‐containing ArfGAP family protein with reported roles in nucleocytoplasmic transport and vesicle/RNA trafficking [16, 17]. In the present case, the breakpoint fell within the N‐terminal ArfGAP domain. Therefore, only part of the ArfGAP domain was predicted to be retained, whereas the remainder of that domain and the entire downstream FG‐rich region were lost. Because the *ERBB4* contribution begins at exon 2, downstream of the native signal peptide, translation was predicted to initiate at the *AGFG1* start codon. We compared the present case with the only *ERBB4* fusion identified in the TCGA cutaneous melanoma cohort, *CPSF4L*::*ERBB4* in TCGA‐ER‐A194 [11]. Despite different 5′ partners, both share an *ERBB4* exon 2 breakpoint. We hypothesize that the retained *AGFG1* moiety may contribute to aberrant, ligand‐independent *ERBB4* activation through oligomerization or disruption of normal receptor autoinhibition. However, *AGFG1* is not an established dimerization partner; therefore, this remains speculative and requires functional validation.

Elenius and colleagues have demonstrated that the oncogenicity of *ERBB4* mutant alleles is isoform‐specific [10]. In this tumor, the *ERBB4* breakpoint in exon 2 lies upstream of both major alternatively spliced regions that generate the JM‐a/JM‐b and CYT‐1/CYT‐2 isoforms [7]. Therefore, the breakpoint itself does not define the downstream *ERBB4* splice isoform. Although the fusion was detected by whole transcriptome RNA sequencing, the available clinical report does not provide sufficient information to resolve the complete JM/CYT splice configuration. Because the biologic effects of *AGFG1*::*ERBB4* may be isoform‐dependent, this limitation warrants further study.

Although canonical Spitz‐associated kinase fusions involve *ROS1*, *ALK*, *NTRK1–3*, *MET*, *RET*, *BRAF*, and *MAP3K8*, *ERBB4* has been documented as a rare, noncanonical fusion partner within the Spitz spectrum [18, 19]. Quan et al. identified a previously unreported *MYO5A*::*ERBB4* fusion in a Spitz tumor, expanding the recognized repertoire of kinase fusions in these lesions [20]. However, the presence of an *ERBB4* fusion is not sufficient to assign Spitz lineage, as fusion partner, morphology, and the broader molecular background must be interpreted together. Kinase gene fusions were historically regarded as markers of Spitz lineage, but they are now well documented in bona fide conventional melanomas of non‐Spitz lineage, including aggressive fusion‐driven tumors lacking spitzoid cytomorphology [6, 21, 22, 23, 24]. The present tumor harbored a pathogenic *NF1* loss‐of‐function alteration, characteristic of conventional, non‐Spitz melanoma rather than of Spitz neoplasia. Taken together, the morphological and molecular findings support a diagnosis of conventional non‐Spitz melanoma and argue against a Spitz lineage.

In the present case, the co‐occurrence of *ERBB4* fusion with *NF1* loss is particularly notable because recent studies have linked *NF1*‐mutant melanoma to ERBB‐family receptor activation [25]. *NF1* loss increases RAS‐GTP levels and promotes constitutive RAS–MAPK pathway activation [5]. Beyond this canonical MAPK effect, *NF1* loss has been shown to increase EGFR/ERBB1 expression and activation in melanoma, resulting in activation of both the RAS‐MAPK and PI3K‐AKT signaling pathways and sensitivity to EGFR inhibition in *NF1*‐mutant melanoma models [25]. Because ERBB4 can activate both pathways, *AGFG1::ERBB4* may provide an additional upstream ERBB‐family signaling input, whereas *NF1* loss drives downstream RAS‐MAPK pathway dependence and may promote broader ERBB‐family signaling vulnerability. This molecular context supports the possibility that *AGFG1::ERBB4* functions as a cooperating event in *NF1*‐mutant melanoma.

Gene fusions are rare in melanoma but may represent clinically actionable genomic alterations [6]. A distinctive biologic feature of *ERBB4* is that its intracellular domain can undergo proteolytic cleavage and translocate to the nucleus, where it may influence gene transcription [26]. The retention of the *ERBB4* kinase domain raises the possibility of both broader ERBB‐family signaling and altered transcriptional regulation, providing a structural rationale for potential sensitivity to pan‐ERBB inhibition, although these possibilities require validation. Preclinical data suggest that *NF1*‐mutant melanomas do not respond consistently to single‐agent MEK, ERK, or PI3K‐AKT signaling inhibition [27]. MEK inhibitor monotherapy may also be limited by adaptive ERBB‐family signaling, including upregulation of ERBB3/ERBB2 signaling in *BRAF/NRAS*‐wild‐type melanoma [28]. Consistent with this, *ERBB*‐activated driver‐negative melanoma models show suppression of both RAS‐MAPK and PI3K–AKT signaling with afatinib, and combined afatinib plus trametinib produces stronger antiproliferative effects than either agent alone [29]. Thus, a pan‐ERBB inhibitor could theoretically suppress both fusion‐associated ERBB4 signaling and *NF1*‐loss‐associated ERBB‐family activation, whereas a MEK inhibitor could target downstream RAS‐MAPK pathway dependence. This molecular configuration provides a conceptual rationale for combined pan‐ERBB and MEK inhibition, supporting further investigation of this combination strategy.

## 4 Conclusion

In summary, to our knowledge, this is the first reported *AGFG1::ERBB4* fusion in cutaneous melanoma. This case illustrates the value of integrating histopathologic analysis with DNA‐ and RNA‐based molecular profiling to identify rare but potentially actionable gene fusions in melanoma. Additional cases and functional studies will be needed to determine whether receptor‐preserving *ERBB4* fusions confer sensitivity to pan‐ERBB inhibition.

## Author Contributions

X.C. contributed to study conception and design, clinicopathologic and genomic data review, literature review, figure/table preparation, and manuscript drafting. H.Y, K.L.M., L.L., Y.P., and Y.T.H. contributed to pathologic review, clinicopathologic interpretation and molecular/genomic interpretation. R.M.J. contributed to literature review and data organization. C.C.Y. analyzed the genomic data and critically reviewed the manuscript. S.Z. supervised the study and contributed to study conception and design. All authors critically revised the manuscript and approved the final manuscript.

## Funding

The authors have nothing to report.

## Ethics Statement

The study was performed in accordance with the Declaration of Helsinki. The case was reviewed under Baylor College of Medicine IRB protocol H‐58097, “The Impact of Genomic Profiling and Biomarker Testing on the Diagnosis and Management of Malignancies and/or Rare Diseases.”

## Consent

Informed consent was waived due to the retrospective nature of the study and the use of de‐identified archival material.

## Conflicts of Interest

The authors declare no conflicts of interest.

## Data Availability

The datasets generated and analyzed during the current study are available from the corresponding author upon reasonable request.
